# The m6A reader YTHDF1 facilitates nasopharyngeal carcinoma proliferation and migration *via* c-MYC

**DOI:** 10.1016/j.jbc.2025.110833

**Published:** 2025-10-28

**Authors:** Ping Han, Zhiwen Xiao, Tianliang Xia, Taowei Wu, Shibing Li, Yuchu Ye, Haicang Zeng, Musheng Zeng, Qian Zhong, Xiaoming Huang

**Affiliations:** 1 Department of Otolaryngology-Head and Neck Surgery, Sun Yat-sen Memorial Hospital, Sun Yat-sen University, Guangzhou, China; 2 Guangdong Provincial Key Laboratory of Malignant Tumor Epigenetics and Gene Regulation, Sun Yat-sen Memorial Hospital, Sun Yat-sen University, Guangzhou, China; 3 Department of Otorhinolaryngology Head and Neck Surgery, Biomedical Innovation Center, The Sixth Affiliated Hospital, Sun Yat-sen University, Guangzhou, China; 4 State Key Laboratory of Oncology in South China, Collaborative Innovation Center for Cancer Medicine, Guangdong Key Laboratory of Nasopharyngeal Carcinoma Diagnosis and Therapy, Sun Yat-sen University Cancer Center, Guangzhou, China

**Keywords:** c-MYC, m6A modification, nasopharyngeal carcinoma, mRNA stability, translational regulation, YTHDF1

## Abstract

N6-methyladenosine (m6A), the most prevalent mRNA modification in eukaryotes, is critical for posttranscriptional regulation. The m6A reader YTH domain-containing family protein 1 (YTHDF1) acts as an oncogene in multiple malignancies, yet its specific roles and regulatory mechanisms in nasopharyngeal carcinoma (NPC) have not been fully elucidated. This study elucidates a novel epitranscriptional axis whereby YTHDF1 drives NPC progression *via* posttranscriptional regulation of c-MYC. Clinical analyses reveal YTHDF1 is overexpressed in a subset of NPC specimens, with high expression correlating with advanced stage and poor patient outcomes, supporting its potential as a prognostic biomarker. Functional assays demonstrate that YTHDF1 enhances NPC proliferation, migration, and invasion *in vitro*, while promoting tumor growth and metastasis *in vivo*. Mechanistically, m6A RNA immunoprecipitation sequencing identifies a conserved m6A modification peak within the 3′ untranslated region near the stop codon of c-MYC mRNA, distinct from those reported in other malignancies, that is specifically recognized by YTHDF1, as validated by RIP-qPCR. Unlike its role in other cancers, where YTHDF1 primarily stabilizes c-MYC mRNA, in NPC it exerts dual posttranscriptional control: prolonging c-MYC mRNA half-life to enhance stability and increasing translational efficiency (confirmed by ribosome profiling) to elevate c-MYC protein levels, forming a coordinated regulatory network. Luciferase reporter assays confirm the functional necessity of this m6A site, as mutations abrogate YTHDF1-mediated regulation. Importantly, the m6A inhibitor STM2457 reverses YTHDF1-driven oncogenic phenotypes. These findings uncover a novel mechanism by which YTHDF1 regulates c-MYC through combined effects on mRNA stability and translation, advancing understanding of m6A-mediated oncogenesis and offering new insights into epitranscriptional control of cancer progression.

Nasopharyngeal carcinoma (NPC) arises from nasopharyngeal epithelial cells (NPECs) and has high prevalence rates in and other nations of Southeast Asia. Radiotherapy is considered the standard treatment for NPC because of its radiosensitivity. Local recurrence and distant metastasis are both leading causes of death for patients with NPC. Identifying uncharacterized molecular markers is essential for the development of targeted molecular therapies and precision medicine.

N6-methyladenosine (m6A), the most common and prevalent RNA modification in eukaryotes, has been reported to regulate the stability and translation of mRNAs (1, 2). Abnormal m6A modifications are closely related to tumorigenesis, glycolysis (3), apoptosis, stress granule formation (4) and other biological processes. m6A modifications are dynamically regulated by the methyltransferase complex (m6A writer), demethylases (m6A erasers) and recognition proteins (m6A readers). Recognition proteins usually contain a YTH domain (YT521-B homology), which mediates specific RNA binding abilities and regulates downstream molecular mechanisms accordingly (5). YTH domain-containing family protein 1 (YTHDF1), an m6A reader, enhances translation efficiency by binding to m6A-modified sites. Recent studies have highlighted its prominent roles in regulating tumor proliferation, iron metabolism (6), immune infiltration (7), as well as cancer cell metastasis and chemoresistance (4, 5, 6, 8). YTHDF1 is closely related to the poor clinical prognosis of diverse malignant tumors (9, 10, 11, 12). In hepatocellular carcinoma and gastric carcinogenesis, it facilitates the progression of cancer cells by increasing FZD5 (13) and FZD7 (14) mRNA translation, respectively. YTHDF1 binding in dendritic cells could promote the translation of lysosomal proteases to regulate the immune response (15). Despite the known involvement of m6A modification in tumorigenesis across multiple malignancies, the biological mechanisms underlying YTHDF1-mediated regulation of NPC remain largely unclear.

c-MYC, a recognized oncogene encoding a nuclear phosphorylation protein, regulates cell proliferation, apoptosis, and differentiation (16, 17). Recent evidence has shown that the expression of c-MYC is closely related to the m6A modification level of its mRNA (18, 19, 20, 21, 22, 23). Nevertheless, little is known about the m6A modification of c-MYC RNA in NPC. In this study, we focused on the expression and function of YTHDF1 and reported that it was highly expressed in approximately 49.11% of NPC patients. We further investigated the role of YTHDF1 in NPC tumorigenesis and metastasis. Moreover, we identified c-MYC as an important target gene of YTHDF1 and discovered the m6A sites of MYC mRNA. Taken together, our findings might shed new light on more effective clinical therapies for patients with NPC.

## Results

### High YTHDF1 expression in NPC tissues is related to poor prognosis

**Figure 1:**
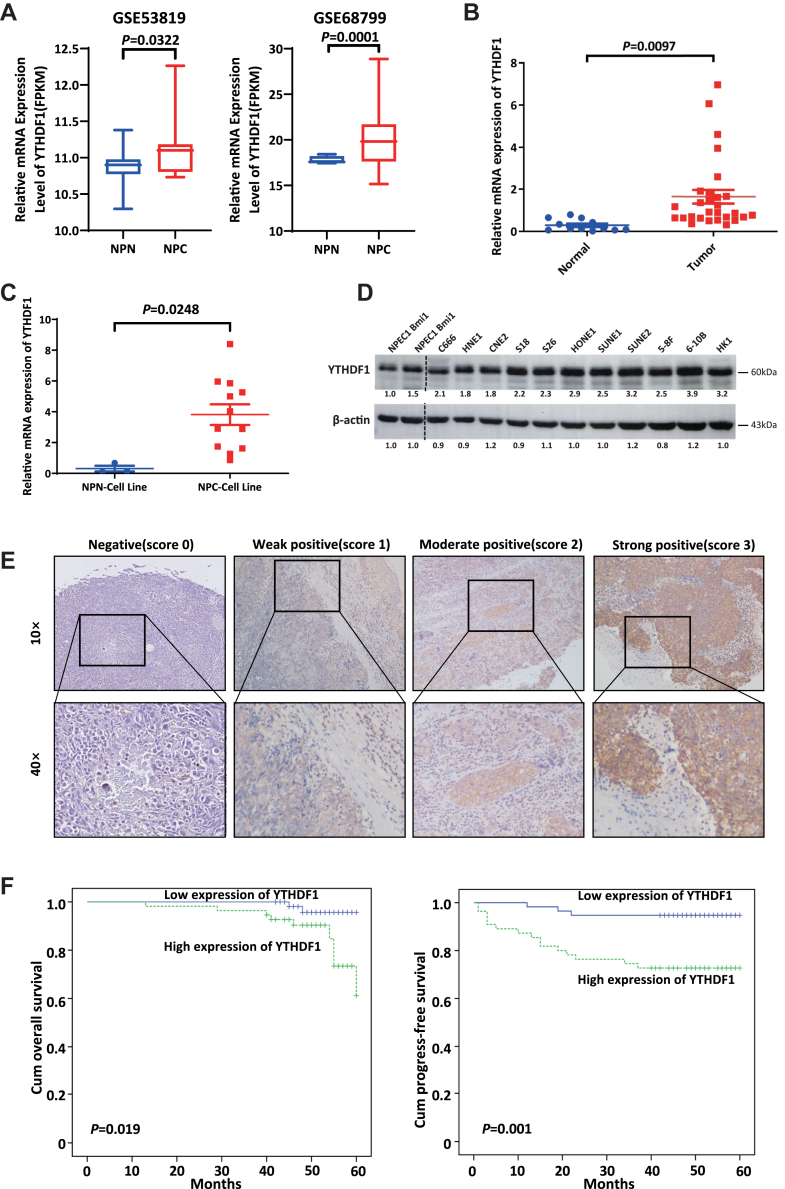
**High YTHDF1 expression in NPC tissues is related to poor prognosis.***A*, box diagrams comparing the mRNA levels of YTHDF1 in NPC *versus* NPN tissues from GSE53819 and GSE68799 datasets; *B*, scatter plots showing YTHDF1 mRNA levels of in 12 NPN and 28 NPC clinical samples; *C*, relative YTHDF1 mRNA levels in immortalized human NPECs and NPC cell lines; *D*, Western blotting analysis of the YTHDF1 protein in NPECs and NPC cell lines; *E*, representative images of immunohistochemical staining for YTHDF1 in NPC tissues (four samples; insets show high-magnification views); (*F*). Kaplan–Meier survival curves for overall survival (*left*) and progression-free survival (*right panel*) of 112 NPC patients, stratified by YTHDF1 IHC scores. IHC, Immunohistochemistry; NPEC, nasopharyngeal epithelial cell; NPC, nasopharyngeal carcinoma; NPN, nasopharyngeal noncancerous; YTHDF1, YTH domain-containing family protein 1.

To investigate the YTHDF1 expression level in NPC and nasopharyngeal noncancerous (NPN) tissues, we analyzed the Gene Expression Omnibus public datasets GSE53819 and GSE68799. These datasets revealed significantly higher YTHDF1 mRNA levels in NPC tissues than in NPN tissues (Fig. 1*A*). In our cohort (28 NPC samples and 12 NPN samples), YTHDF1 mRNA was consistently upregulated in NPC (Fig. 1*B*). Furthermore, YTHDF1 was overexpressed at both the mRNA (Fig. 1*C*) and protein levels (Fig. 1*D*) in most NPC cell lines. In addition, compared with those in NPN, the methyltransferases METTL3 (*p* = 0.0367) and METTL14 (*p* = 0.0043) were significantly upregulated, whereas the demethylases FTO (*p* < 0.0001) and ALKBH5 (*p* < 0.0001) were significantly downregulated (Fig. S1).

To determine the relationship between YTHDF1 expression and clinical outcomes in patients with NPC, Immunohistochemistry (IHC) was performed on samples from 112 individuals (Fig. 1*E*). YTHDF1 shows a trend of high expression in a subset of NPC specimens (49%). High YTHDF1 staining intensity was significantly correlated with poor overall survival (OS, *p* = 0.019) and poor progression-free survival (PFS, *p* = 0.001) in patients with NPC (Fig. 1*F*). Stratification analysis revealed associations between increased YTHDF1 expression and T stage, M stage, and clinical stage (all *p* < 0.05, Table 1). However, no significant correlations were detected between YTHDF1 expression and age, sex, N stage or local recurrence (all *p* > 0.05, Table 1). Univariate analysis identified YTHDF1 as a prognostic factor (*p* = 0.005 for PFS), but multivariate analysis did not confirm its independence (*p* = 0.322) (Table 2). Previous studies have found that NPC patients with distant metastasis have a poorer prognosis (24, 25). These findings suggest that YTHDF1 promotes NPC progression and influence the OS and PFS of NPC patients by enhancing distant metastasis.

**Table 1:** Correlation between YTHDF1 expression and the clinical characteristics of patients with NPC

Characteristic	Patients (n = 112)	Expression of YTHDF1		*pValue*
		Low (%)	High (%)	
Age				0.452
≤47	57	27 (47.4)	30 (54.5)	
>47	55	30 (52.6)	25 (45.5)	
Gender				0.415
Male	88	43 (75.4)	45 (81.8)	
Female	24	14 (24.6)	10 (18.2)	
T Stage				0.011^a^
T1-3	85	49 (86.0)	36 (65.6)	
T4	27	8 (14.0)	19 (34.5)	
N Stage				0.690
N0	9	4 (7.0)	5 (9.1)	
N1/2/3	103	53 (93.0)	50 (90.9)	
M Stage				0.003^a^
0	104	57 (100)	47 (85.5)	
1	8	0 (0)	8 (14.5)	
Clinical stage				0.044^a^
I	4	2 (3.5)	2 (3.6)	
II	16	9 (15.8)	7 (12.7)	
III	60	37 (64.9)	23 (41.8)	
IV	32	9 (15.8)	23 (41.8)	
Local recurrence				0.121
No	104	55 (96.5)	49 (89.1)	
Yes	8	2 (3.5)	6 (10.9)	
Distant metastasis				0.002^a^
No	97	55 (96.5)	42 (76.4)	
Yes	15	2 (3.5)	13 (23.6)	

a *p* value < 0.05.

**Table 2:** Univariate and multivariate Cox proportional hazards analyses for predicting the overall survival and progression-free survival of patients with NPC.

Prognostic factor	Univariate analysis			Multivariate analysis		
	HR	CI	*p Value*	HR	CI	*p Value*
overall survival						
Gender	0.033	0.001–14.198	0.271	…	…	…
Age	1.012	0.949–1.080	0.712	…	…	…
T	1.412	0.715–2.788	0.320	…	…	…
N	0.956	0.451–2.025	0.906	…	…	…
M	6.550	1.726–24.863	0.006^a^	0.349	0.032–3.816	0.388
Local recurrence	3.852	1.007–14.729	0.049^a^	0.485	0.046–5.086	0.546
Distant metastasis	14.505	4.217–49.894	<0.001^a^	21.535	2.589–179.116	0.005^a^
YTHDF1 expression	5.183	1.117–24.047	0.036^a^	2.699	0.454–16.042	0.275
progression-free survival						
Gender	0.033	0.001–3.543	0.153	…	…	…
Age	0.997	0.950–1.046	0.905	…	…	…
T	1.566	0.846–2.896	0.153	…	…	…
N	1.384	0.724–2.643	0.325	…	…	…
M	51.613	17.817–149.512	<0.001^a^	25.369	2.686–239.588	0.005^a^
Local recurrence	10.712	4.080–28.121	<0.001^a^	12.214	1.531–97.488	0.018^a^
Distant metastasis	135.951	29.165–633.731	<0.001^a^	30.394	3.434–269.025	0.002^a^
YTHDF1 expression	5.888	1.704–20.348	0.005^a^	0.325	0.035–3.007	0.322

a *p* value < 0.05.

Taken together, these results indicate that YTHDF1 is upregulated in NPC specimens relative to NPN tissues and is correlated with poor prognosis in advanced cancer and NPC patients.

### YTHDF1 promotes NPC cell proliferation and migration

**Figure 2:**
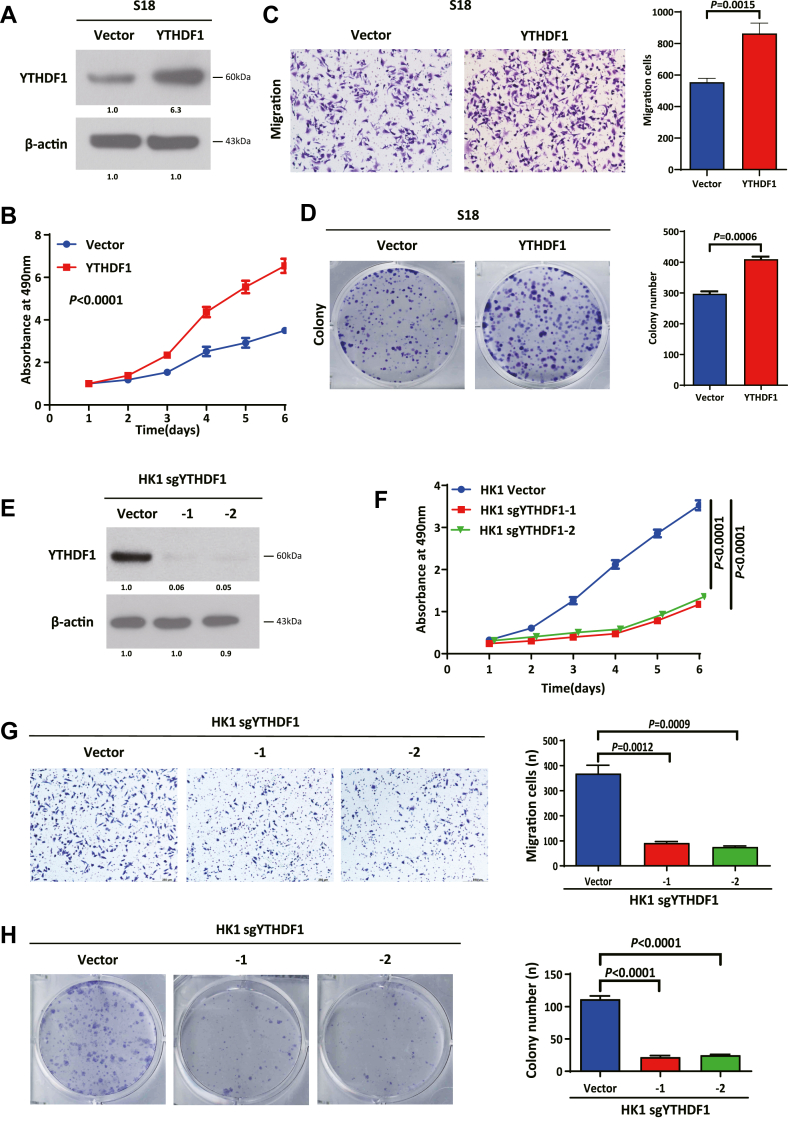
**YTHDF1 promotes NPC cell proliferation and migration.***A*, Western blotting confirming YTHDF1 overexpression in stable YTHDF1-overexpressing S18 cells; *B*, MTT assays showing enhanced proliferation of the YTHDF1-overexpressing S18 cells; *C*, transwell assays demonstrating increased migration capacity of S18 cells with YTHDF1 overexpression; *D*, colony formation assays revealing enhanced clonogenic potential of YTHDF1-overexpressing S18 cells; *E*, Western blotting verifying YTHDF1 KO efficiency in stably edited HK1 cells (using two distinct sgRNAs); *F*, MTT assays showing reduced proliferation of YTHDF1-KO HK1 cells; *G*, transwell assays demonstrating impaired migration of YTHDF1-KO HK1 cells; *H*, colony formation assays revealing decreased clonogenic potential of YTHDF1-KO HK1 cells. Data are presented as means ± SD. Statistical significance was determined using Student's *t* test. YTHDF1, YTH domain-containing family protein 1.

To investigate the function of YTHDF1 in NPC, we generated S18 cells stably overexpressing YTHDF1 (Fig. 2*A*). The MTT assay revealed that YTHDF1 overexpression significantly enhanced S18 cell proliferation (Fig. 2*B*). Transwell and colony formation assays further demonstrated increased migratory (Fig. 2*C*) and clonogenic (Fig. 2*D*) capacities in these cells. Conversely, CRISPR/Cas9-mediated YTHDF1 KO in HK1 cells *via* two distinct sgRNAs (sgYTHDF1-1 and sgYTHDF1-2), confirmed by Western blotting (Fig. 2*E*), resulted in reduced proliferation (Fig. 2*F*), migration (Fig. 2*G*) and colony formation (Fig. 2*H*). Consistent phenotypes were observed in the S26 (Fig. S2, *A*–*D*) and SUNE2 (Fig. S2, *E*–*H*) NPC cell lines. These results collectively indicate that YTHDF1 promotes NPC cell proliferation, migration and clonogenicity.

### YTHDF1 increases c-MYC expression in NPC

**Figure 3:**
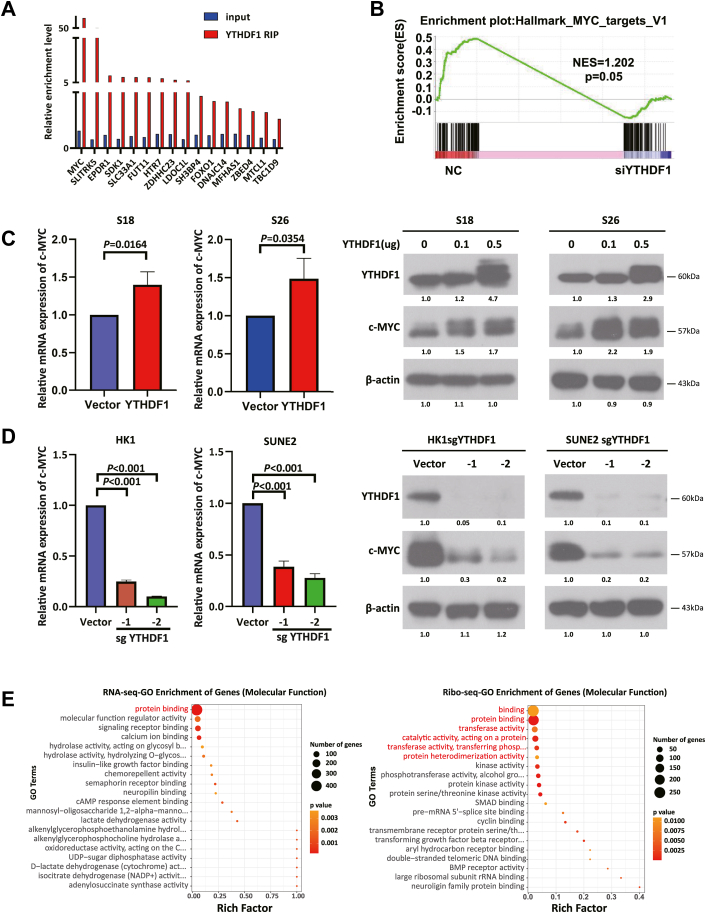
**YTHDF1 upregulates c-MYC expression in NPC.** (*A*). RIP-seq indicating direct binding of YTHDF1 to MYC RNA; *B*, gene set enrichment analysis of the transcriptome sequencing data revealing altered expression of MYC target genes in YTHDF1-KO HK1 cells; *C*, YTHDF1 overexpression significantly increases MYC mRNA levels (*left*) and c-MYC protein expression(*right*) in NPC cell lines in a dose-dependent manner; *D*, YTHDF1 KO significantly reduces MYC mRNA levels (*left*) and c-MYC protein expression (*right*) in NPC cell lines; *E*, molecular function enrichment analysis revealing YTHDF1-associated genes are enriched in protein binding, transferase activity and catalytic activity. YTHDF1, YTH domain-containing family protein 1.

YTHDF1 is a m6A reader that functions by affecting the fate of m6A-modified RNA. To investigate its role in NPC tumorigenesis, RIP assays using YTHDF1-specific antibodies revealed significant enrichment of c-MYC transcripts compared with those in the input controls (Fig. 3*A*). Gene set enrichment analysis was then conducted on the RNA-seq data, screening out the top 10 ranked pathways, including MYC_target_V1 (Figs. 3*B* and S3, *A* and *B*). c-MYC is a well-established driver gene in NPC. Zhao *et al.* reported that METTL3-mediated m6A modification of c-MYC mRNA is recognized by YTHDF1, thereby increasing c-MYC stability (22). In NPC, consistent with this core interaction, YTHDF1 overexpression significantly increased c-MYC mRNA and protein levels in a dose-dependent manner (Fig. 3*C*). However, distinct from their findings, our data further demonstrated that YTHDF1 exerts a more comprehensive regulatory effect on c-MYC in NPC: the mRNA expression of MYC was higher in the WT cell lines than in the YTHDF1-KO cell lines, and c-MYC expression decreased in the stable YTHDF1-KO cell lines (Fig. 3*D*), suggesting that YTHDF1-mediated regulation is not limited to stability but involves multilevel modulation. Additionally, ribosome profiling (Ribo-seq) was performed in YTHDF1-knockdown (n = 3) and control HK1 cells (n = 3). GO enrichment analysis of molecular function revealed that it was significantly enriched in protein binding, transferase activity and catalytic activity (Fig. 3*E*).

Collectively, these data demonstrate that YTHDF1 could posttranscriptionally upregulate c-MYC in NPC.

### YTHDF1 enhances c-MYC translation *via* m6A-dependent mechanisms

**Figure 4:**
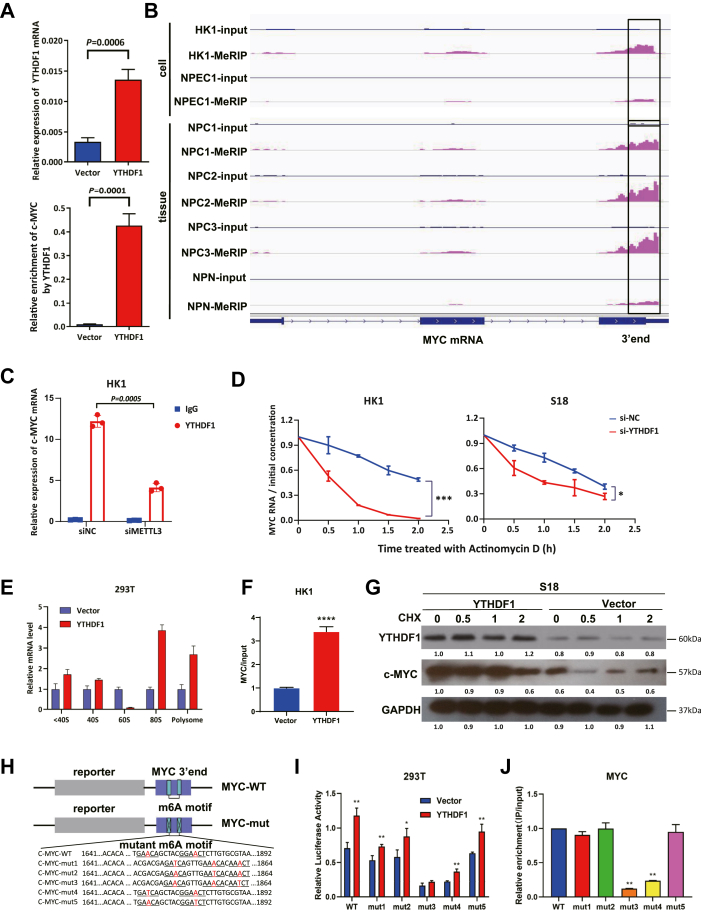
**YTHDF1 enhances c-MYC translation *via* m6A-dependent mechanisms.***A*, RIP-qPCR showing significantly higher enrichment of c-MYC mRNA in YTHDF1-overexpressing cells compared to controls (*p* = 0.0001); *B*, m6A RNA immunoprecipitation sequencing analysis of HK1 cells, NPEC1/Bmi1 cells, 3 NPC tissues and 1 NPN tissue revealing enriched m6A modifications near the stop codon of c-MYC mRNA; *C*, RIP-qPCR demonstrating reduced YTHDF1-mediated enrichment of MYC mRNA in METTL3-knockdown cells (*p* = 0.0005); *D*, quantitative real-time polymerase chain reaction analysis after Actinomycin D treatment, showing significantly shortened MYC mRNA half-life and reduced stability in YTHDF1-KO HK1 and S18 cells (∗*p* < 0.05, ∗∗∗*p* < 0.001); *E*, Ribosome profiling results indicating increased MYC mRNA enrichment in the translation pool of ribosomes upon YTHDF1 overexpression; *F*, YTHDF1 overexpression significantly increases the MYC/input ratio in polysome fractions of HK1 cells (∗∗∗∗*p* < 0.0001); *G*, cycloheximide chase assay demonstrating prolonged c-MYC protein half-life in YTHDF1-overexpressing in S18 cells. *H*, schematic of luciferase reporter constructs: WT PGL3-c-MYC-WT (c-MYC-WT) and the mutant m6A motif PGL3-c-MYC-mut (c-MYC-mut). *I*, Luciferase assay revealing that YTHDF1 increased the luciferase activity of WT c-MYC (c-MYC-WT); however, the luciferase activity decreased after one-by-one mutation of the c-MYC m6A motif (∗*p* < 0.05, ∗∗*p* < 0.01); *J*, RIP-qPCR showing decreased binding of YTHDF1 to c-MYC mRNA harboring mutations in mutant 3 and 4 c-MYC in HEK-293T cell line (∗∗*p* < 0.01). YTHDF1, YTH domain-containing family protein 1.

As a m6A reader, YTHDF1 promotes the translation and stability of methylated transcripts (12, 22). RIP-qPCR confirmed significant enrichment of c-MYC mRNA in the YTHDF1-overexpressing cells compared with the control cells (Fig. 4*A*), which aligns with the previous RNA immunoprecipitation sequencing data and indicates that YTHDF1 binds to m6A sites on c-MYC. m6A RNA immunoprecipitation sequencing analysis of HK1 cells and three NPC tissue samples revealed elevated m6A modification peaks near the c-MYC stop codon relative to those in controls (Fig. 4*B*).

MYC, located on human chromosome 8, is a key proto-oncogene (26). Its mRNA contains multiple m6A sites within the coding region determinant (CRD), which can be recognized by m6A readers to increase mRNA stability and translation (27). To determine how YTHDF1 regulates c-MYC, Ribo-seq results revealed that YTHDF1 selectively enhances the translation of m6A-modified transcripts, including MYC, without globally altering translational efficiency (TE) (Fig. S4, *A*–*G*). RIP-qPCR revealed that METTL3 knockdown reduced c-MYC enrichment in the YTHDF1 immunoprecipitates (*p* = 0.0005, Fig. 4*C*), indicating that METTL3-dependent m6A modification is required for YTHDF1 binding. In YTHDF1 overexpressing cells, nuclear fractionation assays combined with m6A inhibition (STM2457) revealed that reduced m6A modification significantly impaired the nuclear export of c-MYC (Fig. S5*A*), indicating that m6A modification may be involved in regulating c-MYC nuclear export in this context. However, whether YTHDF1 directly mediates this process requires further investigation. RNA stability assays further revealed that YTHDF1 knockdown shortened the MYC mRNA half-life (Fig. 4*D*). Concurrent with enhanced stability, polysome profiling revealed that compared with the control, YTHDF1 overexpression enriched MYC mRNA in the translated ribosomal fractions, including 80S monosomes and polysomes (Fig. 4, *E* and *F*), indicating enhanced ribosomal engagement. To assess c-MYC protein turnover, control and YTHDF1-overexpressing HK1 or S18 cells were treated with Cycloheximide (CHX) (5 μg/ml) and harvested at 0, 0.5, 1 and 2 h. Western blotting analysis revealed that YTHDF1 overexpression significantly prolonged the c-MYC half-life (Figs. 4*G* and S5*B*).

To map functional m6A sites, we generated luciferase reporters containing either the WT 252 nt c-MYC CRD fragment or mutants with A to T substitutions at five predicted m6A motifs (Fig. 4*H*). The luciferase activities of the mutant 3 and mutant 4 c-MYC reporters were significantly decreased (Fig. 4*I*), and anti-YTHDF1 RIP-qPCR analysis revealed reduced YTHDF1 binding to these mutants of c-MYC mRNA (Fig. 4*J*). These results indicate that m6A motif mutants 3 and 4 (corresponding to the third and fourth predicted m6A sites in the c-MYC CRD) are critical for YTHDF1-mediated translational regulation, demonstrating that YTHDF1 enhances c-MYC expression by recognizing m6A modifications at specific CRD sites in NPC.

Taken together, these findings indicate that YTHDF1 can bind to m6A-modified MYC mRNA to promote its TE and stabilize the resulting c-MYC protein, thereby increasing c-MYC expression in NPC cells.

### YTHDF1 drives NPC malignancy *via* m6A-dependent c-MYC regulation

**Figure 5:**
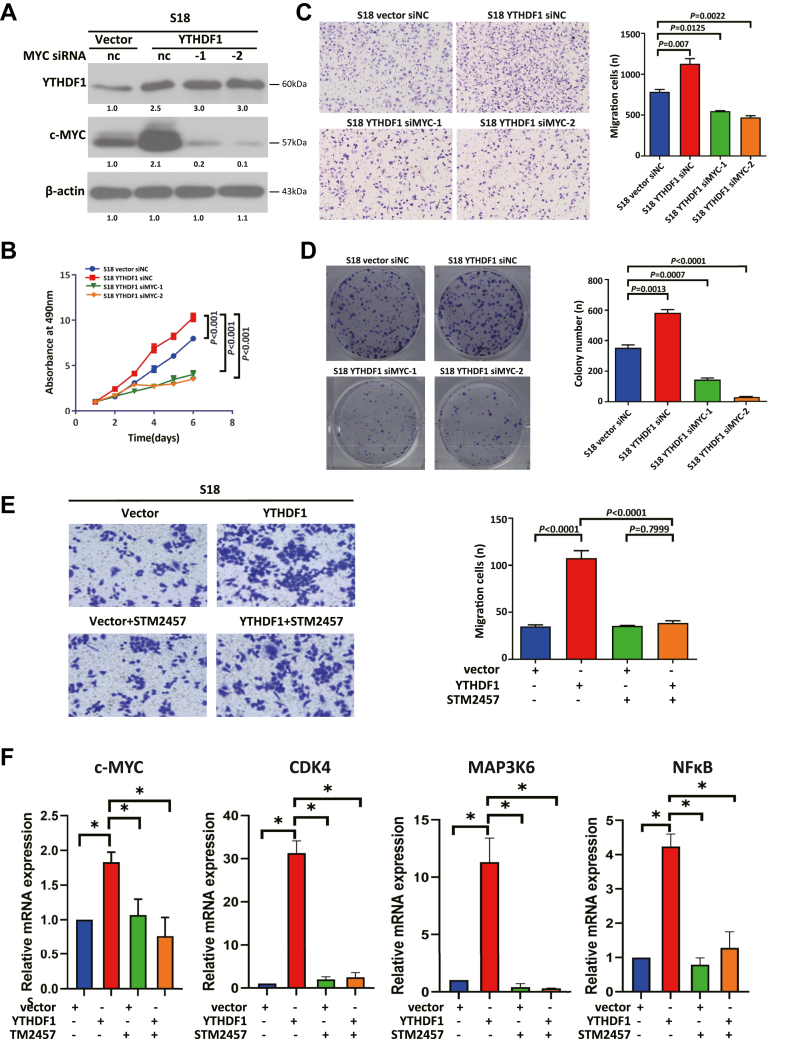
**YTHDF1 drives NPC malignancy *via* m6A-dependent c-MYC regulation.***A*, verification of siRNA-mediated c-MYC knockdown efficiency in YTHDF1-overexpressing S18 cells; *B*, MTT assay showing abrogated proliferation of YTHDF1-overexpressing S18 cells upon c-MYC silencing; *C*, transwell assay revealing impaired migration of YTHDF1-overexpressing S18 cells after c-MYC knockdown; *D*, colony formation assays revealing reduced clonogenic potential of YTHDF1-overexpressing S18 cells upon c-MYC silencing; *E*, the m6A-specific inhibitor STM2457 partially reverses YTHDF1-induced migratory capacity; *F*, STM2457 alters mRNA expression of c-MYC downstream genes (∗*p* < 0.05). YTHDF1, YTH domain-containing family protein 1.

Given the role of YTHDF1 in the upregulation of c-MYC, we investigated whether this axis mediates NPC malignancy. By using c-MYC siRNA in stable YTHDF1-overexpressing cell lines, we observed that c-MYC expression was significantly downregulated (Fig. 5*A*), indicating that YTHDF1 is an upstream regulator. Functional assays revealed that c-MYC depletion abrogated YTHDF1-driven proliferation, migration, and colony formation (Fig. 5, *B*–*D*), with consistent results in an independent NPC cell line (Fig. S6, *A*–*D*).

To evaluate YTHDF1 as a therapeutic target, we performed functional experiments using the m6A-specific inhibitor STM2457. STM2457 partially reversed YTHDF1-induced migration (Fig. 5*E*) and normalized the expression of c-MYC downstream targets (Fig. 5*F*). These findings suggest that YTHDF1-dependent m6A modification promotes NPC aggressiveness and that STM2457 can reverse these effects.

### YTHDF1 upregulates c-MYC expression in specimens from clinical specimens and in *invivo* models

**Figure 6:**
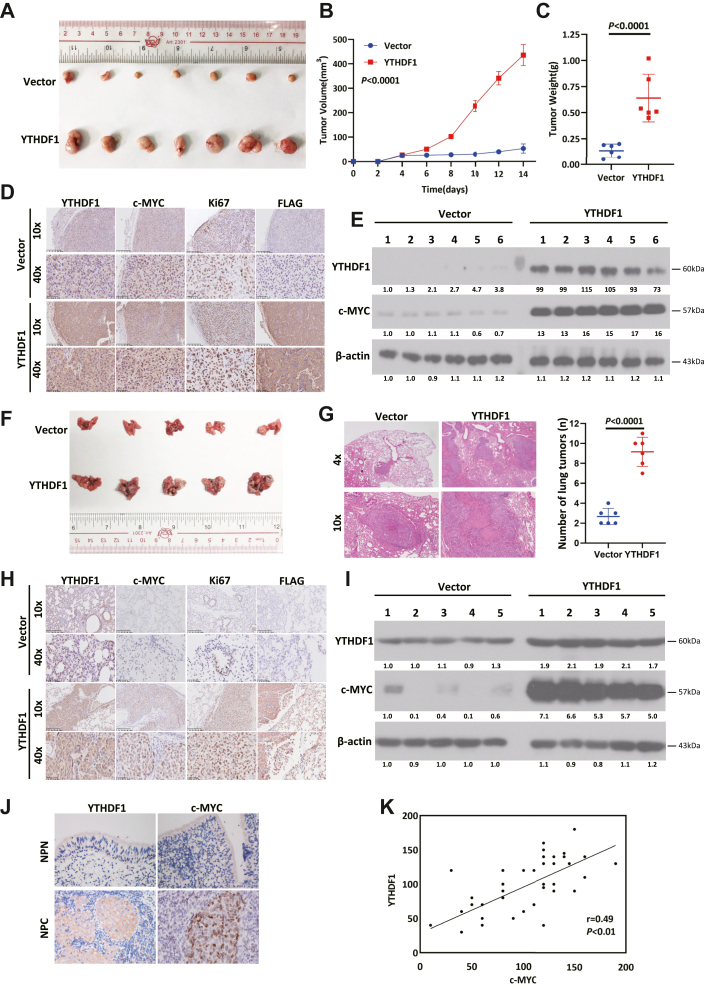
**YTHDF1 upregulates c-MYC in clinical specimens and *in vivo* models.***A*, YTHDF1 overexpression significantly accelerates tumor growth in mice. Tumor volume was measured every 2 days, and growth curves are shown; *B*, tumors were dissected from nude mice at 14 days post-implantation, photographed, and weighed; *C*, schematic of subcutaneous implantation of YTHDF1-overexpressing or control cells into nude mice; *D*, IHC analysis of xenograft tumors showing strong expression of YTHDF1, c-MYC, and Ki67 in the YTHDF1-overexpressing group; FLAG-tag (exogenous YTHDF1) was detected only in the overexpression group; *E*, Western blotting analysis of xenograft tumors showing elevated expression of YTHDF1 and c-MYC in the YTHDF1-overexpressing group relative to controls; *F*, YTHDF1 overexpression of significantly increased tumor cell metastasis to mouse lungs. Nude mice were injected *via* the tail vein with either YTHDF1-overexpressing cells or empty vector-transfected control cells, with a greater number of metastatic lung tumors observed in the YTHDF1-overexpressing group; *G*, representative images of H&E -stained metastatic lung tumors (*left panel*). Quantification of metastatic lung tumors derived from YTHDF1-overexpressing and control S18 cells (right panel). Data are presented as the means ± S.D, with statistical significance determined using a two-sided Student’s *t* test; *H*, IHC analysis of lung metastasis tissues revealing increased expression of YTHDF1, c-MYC, and Ki67 in the YTHDF1-overexpressing group. FLAG-tagged exogenous YTHDF1 was detected exclusively in the overexpression group, with no signal observed in controls; *I*, Western blotting analysis of lung metastasis tissues revealing significantly higher expression levels of YTHDF1 and c-MYC in the YTHDF1-overexpressing group compared to controls; *J*, representative images of IHC staining for YTHDF1 and c-MYC in NPN and NPC tissues; *K*, correlation analysis of YTHDF1 and c-MYC expression levels based on IHC staining, showing a positive association (r = 0.49, *p* < 0.01). IHC, Immunohistochemistry; YTHDF1, YTH domain-containing family protein 1.

To validate the oncogenic potential of YTHDF1 *in* *vivo*, YTHDF1-overexpressing cells were subcutaneously injected into nude mice. Compared with the control group, the overexpression group presented significantly greater tumor volumes and greater masses (*p* < 0.0001, Fig. 6, *A*–*C*). IHC and Western blotting analyses confirmed upregulated YTHDF1 and c-MYC expression in subcutaneous xenografts derived from YTHDF1-overexpressing cells (Fig. 6, *D* and *E*), with stronger FLAG-tag staining (indicating exogenous YTHDF1) in the overexpression group (Fig. 6*D*).

In a tail vein injection model of metastasis, YTHDF1 overexpression increased the number and volume of lung xenograft tumors, as observed macroscopically and microscopically (Fig. 6, *F* and *G*, *p* < 0.0001). Consistent with the findings in the subcutaneous models, the lung xenografts derived from the YTHDF1-overexpressing cells presented increased YTHDF1 and c-MYC expression (Fig. 6, *H* and *I*), with increased FLAG-tag staining (Fig. 6*H*).

To explore their correlation in clinical settings, 46 NPC samples were stained with YTHDF1 and c-MYC antibodies and subjected to IHC analysis. We detected strong staining of both YTHDF1 (in the cytoplasm) and c-MYC (in the nucleus) in NPC tumors, whereas the staining intensities of YTHDF1 and c-MYC were relatively weak in NPN tissues (Fig. 6*J*). In addition, cohigh expression of YTHDF1 and c-MYC was detected in 65.22% (30/46) of the NPC samples, and linear regression analysis confirmed a positive association (r = 0.49, *p* = 0.01) (Fig. 6*K*).

Collectively, these findings demonstrate that YTHDF1 acts as an upstream regulator to promote c-MYC expression in NPC, as validated in both murine models and clinical specimens.

## Discussion

YTHDF1, a key m6A reader (28), acts as an oncogenic driver in multiple cancers by modulating mRNA translation and stability, but its role in NPC, especially its mechanism of regulating critical oncogenes like c-MYC, remains poorly defined. Our study addresses this gap by demonstrating that YTHDF1 promotes NPC proliferation, migration, and metastasis *via* a unique m6A-dependent dual regulation of c-MYC: prolonging mRNA half-life and enhancing TE.

Firstly, we explored the clinical relevance of YTHDF1 in NPC *via* public database mining and IHC analysis. We found that YTHDF1 is upregulated in NPC tissues compared to NPN tissues, and this upregulation was associated with high T and M stages, shorter OS and PFS, and poor prognosis, supporting its potential as a prognostic biomarker. However, the small sample size of in-house cohort (n = 112) may compromise its independent prognostic value, this is reflected in our multivariate analysis, where YTHDF1 did not reach statistical significance as an independent prognostic factor (*p* = 0.322). Notably, the nonsignificant independent prognostic value of YTHDF1 may not be resolved by increasing sample size alone, as it could be inherently confounded by other key prognostic factors in NPC (*e.g.*, distant metastasis) that often coexist with high YTHDF1 expression and exert stronger impacts on patient outcomes. Thus, larger, multi-center studies with well-stratified clinical subgroups (*e.g.*, stratified by metastasis status or clinical stage) are needed to further clarify and confirm the clinical utility of YTHDF1 as a prognostic biomarker in NPC. Functional assays further validated the oncogenic role of YTHDF1. YTHDF1 enhances NPC cell proliferation, migration, and invasion *in vitro* and promotes subcutaneous tumor growth and lung metastasis in *vivo,* which is consistent with its oncogenic role in other malignancies (29) but extends this role to NPC.

Mechanistically, we focused on the YTHDF1-mediated regulation of c-MYC, a master oncogene that governs glycolysis, cell cycle progression, and drug resistance (30, 31). c-MYC mRNA m6A modifications are functionally divergent: the 5′ end sites of c-MYC RNA mainly promote mRNA degradation, whereas the 3′ end sites enhance mRNA stability (32, 33). In esophageal squamous cell carcinoma, YTHDF1 is involved in m6A-dependent regulation, and c-MYC is a key downstream effector in the oncogenic pathway mediated by METTL3/YTHDF-coupled epitranscriptomal regulation (34). However, the study failed to establish a direct m6A-dependent regulatory association between YTHDF1 and c-MYC, thereby leaving room for investigating the divergent regulatory effects of YTHDF1 on c-MYC across different cancers.

Our results revealed a previously uncharacterized regulatory axis in NPC that is distinct from the METTL3-YTHDF1-c-MYC pathway reported in oral squamous cell carcinoma (22). In their study, YTHDF1 was shown to enhance c-MYC stability primarily by recognizing METTL3-mediated m6A modifications with preliminary evidence of translational regulation. However, their study did not explore the coordination of stability and translation. In contrast, our findings in NPC demonstrate that YTHDF1, as a m6A reader, recognizes preexisting m6A sites in the c-MYC CRD (1860-…AAACT…-1864 and 1866-…GAACA…-1870) without altering c-MYC m6A levels. This recognition upregulates c-MYC through three interconnected mechanisms: (1) Act-D chase assays confirmed that YTHDF1 prolongs the c-MYC mRNA half-life, increasing its intracellular accumulation; (2) Polysome profiling revealed that YTHDF1 overexpression enriched c-MYC mRNA in the translated ribosomal fractions, including 80S monosomes and polysomes; Ribo-seq further demonstrated that YTHDF1 specifically enhances the translation of m6A-modified genes (including c-MYC) without affecting global TEy; (3) Reduced m6A (*via* STM2457) impairs c-MYC nuclear export in YTHDF1-overexpressing cells (Fig. S5*A*), suggesting m6A may regulate nuclear export, though YTHDF1’s direct role requires validation. This multi-layered regulation is absent in oral squamous cell carcinoma, where YTHDF1-mediated regulation of c-MYC is limited to mRNA stability without evidence of translational coordination or nuclear export involvement (22). All these regulatory mechanisms are likely interconnected: increased mRNA stability provides more translatable templates, whereas increased ribosomal assembly enhances protein synthesis.

Luciferase reporter assays further identified m6A motif mutants 3 and 4 within the CRD region (1860–1870) as critical for the binding of YTHDF1 to the c-MYC CRD. To our knowledge, these m6A motifs (AAACT and GAACA within the CRD) are unreported in NPC and distinct from the 3′ untranslated region -localized GGACU motif targeted by METTL3/YTHDF1 in oral squamous cell carcinoma (22), highlighting a tissue-specific m6A code for c-MYC regulation. Importantly, mutations at these sites significantly reduce YTHDF1 binding and TE, supporting the unique c-MYC regulatory landscape of NPC. This specificity may explain the positive correlation between YTHDF1 and c-MYC expression in 65.22% of the NPC samples.

Notably, YTHDF1 acts as a canonical m6A reader that regulates mRNA fate (*e.g.*, stability or translation) by recognizing preexisting m6A marks rather than modifying m6A levels. This m6A dependence is validated by two key findings: STM2457 (m6A inhibitor) reversed YTHDF1-induced migration, and c-MYC knockdown abrogated the oncogenic effects of YTHDF1, confirming that c-MYC is a key downstream effector.

Our findings extend existing c-MYC regulation knowledge beyond existing models. METTL3, a m6A writer, enhances c-MYC stability by modifying m6A levels in oral carcinoma (22), whereas YTHDF2, a m6A reader, promotes its degradation (35). In contrast, our study revealed that YTHDF1 in NPC has a more complex; multifaceted regulatory role on c-MYC. YTHDF1 specifically recognizes m6A sites to promote translation and stability, highlighting the distinct c-MYC regulatory landscape of NPC.

Clinically, YTHDF1 is a potential prognostic biomarker, and the ability of STM2457 to reverse YTHDF1-mediated phenotypes supports targeting m6A-dependent pathways in NPC. However, this study has several limitations. First, the patient cohort was relatively small, and validation in larger multicenter studies is needed. Second, translational relevance needs to be confirmed using orthotopic NPC models (*versus* subcutaneous or tail vein injection). Future studies will address these gaps by validating the m6A-dependent regulation of c-MYC (including 3′ untranslated region translational control and m6A level quantification *via* luciferase assays and LC‒MS/MS) and dissecting the cooperative effects of YTHDF1 targets in oncogenic signaling.

In summary, we identify YTHDF1 as an NPC oncogene that promotes progression by m6A-dependent recognition of specific c-MYC CRD sites, enhancing c-MYC translation and stability. These findings advance the understanding of m6A-mediated posttranscriptional control in NPC and provide a preclinical foundation for targeting the YTHDF1-c-MYC axis.

## Experimental procedures

### Tissue specimen collection

From June to September 2018, 28 NPC biopsy samples and 12 NPN biopsy samples were collected from Sun Yat-sen Memorial Hospital, Sun Yat-sen University (SYSMH, SYSU). These samples were analyzed using quantitative real-time polymerase chain reaction. From May 2012 to June 2017, paraffin-embedded tissues from 112 NPC patients (median age: 46 years, range 19–65 years) were obtained at SYSMH, SYSU, for immunohistochemistry analyses.

Ethical committee approval was granted by the ethics committees of SYSMH and SYSU (SYSKY-2023-298-01) and followed the ethical guidelines of the Declaration of Helsinki. The patients with NPC in this study provided informed consent for the use of their specimens and clinical data. The patients’ clinical characteristics are described in Table 1. In addition, all murine experiments were approved by the Ethics Board of Sun Yat-sen University.

### Cell lines and culture

NPC cell lines were obtained from the Sun Yat-sen University Cancer Center (SYUCC). Immortalized human NPECs, including the NPEC2-Bmi1, NPEC1-Bmi1 and NP69 cell lines, were cultured in K-SFM. C666, HNE1, S26, S18, HK1, 5-8F, 6-10B, HONE1, SUNE1, SUNE2 and CNE2 cells were cultured in RPMI-1640 medium supplemented with 5% fetal bovine serum (FBS). The 293T cell line was maintained in Dulbecco's modified Eagle medium (DMEM) supplemented with 10% FBS, streptomycin (200 μg/ml; Gibco) and penicillin (100 U/ml; Gibco). The cells were cultured at 37 °C in a humidified incubator with 5% CO2.

### Plasmids

The YTHDF1-pENTER and c-MYC-pENTER plasmids were purchased from Vigene Biosciences. Plasmids encoding YTHDF1 and c-MYC with myc tags were established by delivering cDNAs into the pcDNA6/myc-His empty vector plasmid, and WT and mutated c-MYC plasmids were established by delivering cDNAs into the pLVX-DsRed-Monomer-N1 empty vector plasmid. sgRNAs targeting YTHDF1 were synthesized, annealed and cloned and inserted into a lentiviral vector to knock out YTHDF1 expression. All primer sequences are provided in Table S1.

### Establishment of cell lines

NPC cells were transfected with transient expression vectors *via* Lipofectamine 3000 (Invitrogen). The lentiviral plasmid was transfected into 293T cells together with the packaging plasmids psPAX2 and pMD2. G using PEI (Invitrogen). Infectious lentivirus particles were harvested 48 h after transfection, filtered through 0.45 μm filters and transduced into NPC cells.

To generate stable YTHDF1-overexpressing cells, NPC cells were infected with packaged virus and selected with puromycin (1 μg/ml) for 3 days. The surviving cells were selected and seeded into culture flasks for the formation of cell clones and further expansion.

### Total RNA extraction and real-time RT–qPCR

Total RNA was extracted from different NPC cells and tissues *via* TRIzol reagent (Invitrogen). According to the manufacturer’s instructions for the reverse transcription kit, reverse transcription was performed to obtain cDNA (Promega). RT–qPCR assays were then used to determine the mRNA levels of YTHDF1 and c-MYC *via* SYBR Green (Roche) and a Roche LC480 instrument (Roche Diagnostics, Switzerland) following the manufacturer’s instructions, with β-actin used as a loading control. The results were subsequently analyzed by calculating the 2^-ΔΔCt^ value of YTHDF1 or MYC relative to that of β-actin. All sequences of primers used in our study are provided in Table S2.

### Western blotting

Western blotting was performed as previously described (36). Band intensities were quantified *via* ImageJ software (https://imagej.nih.gov/ij/), and the relative expression of target proteins was calculated relative to that of β-actin/GADPH. The antibodies used were as follows: anti-YTHDF1 (WB, ab99080, MA, Abcam); anti-c-MYC (IHC, WB, 9402S, MA, CST); anti-YTHDF1 (IHC, 17479-1-AP, ProteinTech, IL); Ki67 (IHC, ab16667, Abcam); anti-ACTB (WB, 66009-1-Ig, ProteinTech); anti-FLAG-tag (IHC, 14793S, CST); and anti-MYC-tag (WB, 16286-1-AP, ProteinTech).

### Migration assay

After 1 × 10^5^ HK1 or 0.5 × 10^5^ S18 cells were inoculated into the upper chamber of a Transwell insert (pore size = 8 μm; Corning Falcon) in 200 μl of RPMI-1640 medium without serum, they were placed into the lower chamber of a Transwell containing 600 μl of RPMI-1640 medium supplemented with 5% FBS. After 18 to 24 h, the cells were fixed in anhydrous methanol and stained with 0.5% crystal violet. Finally, the migrated NPC cells that penetrated through the bottom of the chambers were photographed and counted under a microscope. All the experiments were repeated three times.

### Colony formation assay

To detect the influence of YTHDF1 on NPC cell tumorigenesis, colony formation assays were conducted as previously reported (37). After fixation in anhydrous methanol and staining with 0.5% crystal violet, colonies consisting of ≥50 cells were counted.

### 3-(4,5-Dimethylthiazol-2-yl)-2,5-diphenyltetrazolium Bromide (MTT) assay

The MTT assay was used to evaluate the viability of NPC cells in 96-well plates at 1000 cells/well in 200 μl of RPMI-1640 medium. At the indicated time points, 20 μl of MTT solution (5 mg/ml) was added to each well, followed by incubation at 37 °C for 4 h. The formazan crystals were solubilized by adding 200 μl of dimethyl sulfoxide to dissolve the crystals for 10 min, and the absorbance of each well at 490 nm was measured over the next 6 days (38).

### Immunohistochemistry (IHC)

NPC tissues and NPN tissues were subjected to IHC staining with antibodies against YTHDF1 and c-MYC. IHC for YTHDF1, c-MYC, Ki67 and FLAG was performed on sections from tumor tissues from nude mice. After deparaffinization and antigen retrieval, the tissue sections were incubated with specific primary antibodies against YTHDF1, c-MYC, FLAG and Ki67 at 4 °C overnight, followed by incubation with secondary antibodies at 37 °C for 30 min. The slides were then processed *via* the Envision system and DAB-chromogen, with subsequent hematoxylin counterstaining. Staining was evaluated by two independent pathologists in a blinded manner. The staining intensities were graded as none (0 points), weak (1 point), moderate (2 points) or strong (3 points), as previously reported (38).

### Establishment of nude mouse models for evaluating tumorigenesis and lung metastasis

To assess *in vivo* tumorigenic and metastatic potential, 2 × 10^6^ cells in serum-free RPMI-1640 medium were subcutaneously injected into the flanks of nude mice (4–6 weeks) for tumorigenesis assays. For the metastasis models, 1 × 10^6^ cells in 100 μl of cell suspension were injected *via* the tail veins of nude mice (4–6 weeks). The spirits and diets of each nude mouse, together with their weight, were regularly observed and recorded. All the nude mice were sacrificed after 8 weeks, and their lungs were weighed, photographed and sectioned for IHC assays and H&E staining.

### Luciferase reporter assay

WT and mutant c-MYC and WT reporter plasmids were constructed using the pGL3 control vector plasmid for the luciferase reporter assay; primer sequences are provided in Table S3. HEK293T cells were seeded in 24-well plates, cultured to 70% confluency, and then cotransfected with 100 ng of the pGL3-c-MYC-WT or pGL3-c-MYC-mut luciferase reporter plus 20 ng of pRL-TK for normalization. After 36 h, luciferase activity was measured *via* the Dual-Luciferase Reporter Assay System (Promega) following the manufacturer’s instructions. Relative luciferase activity (firefly/Renilla) was calculated to assess the impact of the m6A site on c-MYC regulation.

### m6A RNA immunoprecipitation sequencing (MeRIP-seq)

Total RNA was extracted *via* TRIzol reagent (Invitrogen). RNA fragmentation and m6A-seq were performed according to the manufacturer’s instructions and a previous article (39). The RNA was fragmented to approximately 150 nt and incubated with anti-m6A antibody-conjugated magnetic beads at 4 °C. The m6A-enriched fragments were subsequently eluted and purified. Library preparation and sequencing were performed by Annoroad Gene Technology.

### RNA immunoprecipitation sequencing (RIP-seq)

The RIP assay was conducted *via* the Magna RIP Quad RNA-binding protein immunoprecipitation kit (Millipore). The cells were cultured in 10 cm dishes, harvested and lysed in RIP lysis buffer. The cell lysates (1 ml of RIP buffer) were incubated at 4 °C for 8 h with protein A/G Dynabeads preconjugated to a YTHDF1 antibody or IgG (as a negative control). The enrichment of YTHDF1-binding mRNAs was assessed by RT–qPCR or submitted for sequencing (Annoroad Gene Technology).

### Ribosome profiling (Ribo-seq)

YTHDF1-KO and control cells were treated with 100 μg/ml CHX at 37 °C for 5 min to arrest ribosomes, followed by RNase I digestion of unprotected mRNA regions. Intact mRNA‒ribosome complexes were isolated and processed for sequencing according to Novogene’s protocol. Sequencing was performed on an Illumina HiSeq 4000 (SE50). The raw reads were filtered to remove contaminants mapped to human rRNAs, snoRNAs, snRNAs, and tRNAs (GENCODE v30). High-quality reads were aligned to the human genome *via* Bowtie2 (v2.3.4.3) with -L 10 for optimal short-read mapping. The translation levels of protein-coding genes were quantified *via* featureCounts (v1.6.4), which accounts for multiple mapping reads and prioritizes the longest overlapping feature. Gene set enrichment analysis was performed on Ribo-seq data to compare translation efficiency between YTHDF1-KO and control cells.

### RNA stability assay

Actinomycin D (ActD) was prepared as a 1 mg/ml stock solution according to the manufacturer’s instructions, aliquoted in the dark, and stored at −30 °C. Aliquots were thawed at 4 °C before use. NPC cells were seeded into 12-well plates (four replicate wells per condition) and cultured to 70 to 80% confluency. The medium was then replaced with fresh medium containing 5 μg/ml ActD, the mixture was mixed thoroughly, and the cells were incubated in the dark. Additional ActD was supplemented at 1 h and 1.5 h posttreatment. At 0.5 h after the final addition, the medium was replaced, and RNA was extracted *via* TRIzol. YTHDF1 mRNA levels were quantified *via* quantitative real-time polymerase chain reaction following reverse transcription.

### Cycloheximide (CHX) assays

To assess protein stability, NPC cells (HK1 and S18) were treated with 5 μg/ml CHX for 0 to 2 h. Total protein was extracted and analyzed by Western blotting as previously described.

### Ribosome‒nascent chain complex qPCR

Ribosome‒nascent chain complex extraction was conducted as previously reported (40). Briefly, the cells were treated with 100 μg/ml cycloheximide for 15 min, washed with ice-cold PBS, and lysed in 2 ml of buffer [1% Triton X-100 in RB buffer (20 mM HEPES-KOH pH 7.4, 15 mM MgCl_2_, 200 mM KCl, 100 μg/ml cycloheximide, 2 mM DTT)]. After 30 min on ice, the lysates were centrifuged at 16,200*g* for 10 min at 4 °C. The supernatants were layered onto 30% sucrose in RB buffer and ultracentrifuged at 185,000*g* for 5 h at 4 °C. The ribosome‒nascent chain complex -RNAs were purified and analyzed *via* RT‒qPCR.

### Statistical analysis

Statistical analysis was conducted using GraphPad Prism 8 (GraphPad Software Inc.; https://www.graphpad.com/scientific-software/prism/). Unpaired two-tailed Student’s t tests were used for comparisons. The quantitative data are presented as the means ± SDs. Kaplan–Meier survival curves were generated, and log-rank tests were applied to compare survival between groups stratified by YTHDF1 expression. Univariate and multivariate analyses were performed to identify variables associated with YTHDF1 expression in NPC patients. A Cox regression model was used to determine the independent prognostic factors for the prognosis of NPC. A *p* value of <0.05 was considered to indicate statistical significance.

## Data availability

All data generated or analyzed during this study are contained within the article.

## Supporting information

This article contains supporting information.

## Conflict of interests

The authors declare that they have no conflicts of interest with the contents of this article.
