# Supplementary material for: The m6A reader YTHDF1 facilitates nasopharyngeal carcinoma proliferation and migration via c-MYC
Source: J Biol Chem. 2025 Oct 28;301(12):110833. doi: 10.1016/j.jbc.2025.110833 (PMC12681820; doi:10.1016/j.jbc.2025.110833)
Supplement: Supplementary Table [file mmc1.docx]

**Supplementary table 1. The YTHDF1-pENTER and c-MYC-pENTER plasmids as follows:**

| **No** | **Gene** | **Sequence (5' - 3')** |
| --- | --- | --- |
| 1 | YTHDF1 | Forward:Ttaagcttggtaccgagctcggatccatgtcggccaccagcgtgga |
|  |  | Reverse:TTTTTGTTCGAATGGGTGACCTCGAGCTTGTTTGTTTCGACTCTGCC |
| 2 | c-MYC | Forward:Ttaagcttggtaccgagctcggatccctggatttttttcgggtagt |
|  |  | Reverse:TTTTTGTTCGAATGGGTGACCTCGAGCCGCACAAGAGTTCCGTAGCT |
| 3 | Sg-YTHDF1-1 | Forward:CACCGAAGCATGTCGGCCACCAGCG |
|  |  | Reverse:AAACCGCTGGTGGCCGACATGCTTC |
| 4 | Sg-YTHDF1-2 | Forward:CACCGGCGGCGGTGCAGAGAACAAA |
|  |  | Reverse:AAACTTTGTTCTCTGCACCGCCGCC |
| 5 | c-MYC WT and mutation forward | TCGAGCTCAAGCTTCGAATTCCTGGATTTTTTTCGGGTAGTGGAAAACCAGCCTCCCG |
|  | c-MYC-WT-reverse | ATGGTGGCGACCGGTGGATCCTTACGCACAAGAGTTCCGTAGCTGTTCAAGTTTGTGTTTCAACTG |
|  | c-MYC-mut3-reverse | ATGGTGGCGACCGGTGGATCCTTACGCACAAGAGTTCCGTAGCTGTTCAAGATTGTGTTTCAACTG |
|  | c-MYC-mut4-reverse | ATGGTGGCGACCGGTGGATCCTTACGCACAAGAGTTCCGTAGCTGATCAAGTTTGTGTTTCAACTG |

**Supplementary table 2. qRT-PCR primers used were as follows:**

| **No** | **Gene** | **Sequence (5' - 3')** |
| --- | --- | --- |
| 1 | YTHDF1 | Forward:ACCTGTCCAGCTATTACCCG |
|  |  | Reverse:TGGTGAGGTATGGAATCGGAG |
| 2 | MYC | Forward:GGCTCCTGGCAAAAGGTCA |
|  |  | Reverse:CTGCGTAGTTGTGCTGATGT |
| 3 | METTL3 | Forward:TTGTCTCCAACCTTCCGTAGT |
|  |  | Reverse:CCAGATCAGAGAGGTGGTGTAG |
| 4 | METTL14 | Forward:AGTGCCGACAGCATTGGTG |
|  |  | Reverse:GGAGCAGAGGTATCATAGGAAGC |
| 5 | FTO | Forward:ACTTGGCTCCCTTATCTGACC |
|  |  | Reverse:TGTGCAGTGTGAGAAAGGCTT |
| 6 | ALKBH5 | Forward:CGGCGAAGGCTACACTTACG |
|  |  | Reverse:CCACCAGCTTTTGGATCACCA |
| 7 | β-actin | Forward: CACCATTGGCAATGAGCGGTTC |
|  |  | Reverse:AGGTCTTTGCGGATGTCCACGT |

**Supplementary table 3. The primer sequences for luciferase reporter assay as follows:**

| **No** | **Gene** | **Sequence (5' - 3')** |
| --- | --- | --- |
| 1 | c-MYC forward | GGGCGGAAAGATCGCCGTGACACACAACGTCTTGGAGCGCCA |
| 2 | c-MYC-WT reverse | GCGGCCGGCCGCCCCGACTCTAGATTACGCACAAGAGTTCCGTAGCTG |
| 3 | c-MYC-mut1 reverse | GCGGCCGGCCGCCCCGACTCTAGATTACGCACAAGAGTTCCGTAGCTGTTCAAGTTTGTGTTTCAACTGATCTCGTCGTTTCCGCAACAAG |
| 4 | c-MYC-mut2 reverse | GCGGCCGGCCGCCCCGACTCTAGATTACGCACAAGAGTTCCGTAGCTGTTCAAGTTTGTGATTCAACTGTTCTCGTCGTTTC |
| 5 | c-MYC-mut3 reverse | GCGGCCGGCCGCCCCGACTCTAGATTACGCACAAGAGTTCCGTAGCTGTTCAAGATTGTGTTTCAACTGTTCTCG |
| 6 | c-MYC-mut4 reverse | GCGGCCGGCCGCCCCGACTCTAGATTACGCACAAGAGTTCCGTAGCTGATCAAGTTTGTGTTTCAACTGTTC |
| 7 | c-MYC-mut5 reverse | GCGGCCGGCCGCCCCGACTCTAGATTACGCACAAGAGATCCGTAGCTGTTCAAGTTTGTG |

**Supplementary Table 4 GSEA-enriched pathways**

| **NAME** | **ES** | **NES** | **NOM p-val** |
| --- | --- | --- | --- |
| HALLMARK_E2F_TARGETS | 0.81220454 | 2.0157657 | 0 |
| HALLMARK_G2M_CHECKPOINT | 0.73965704 | 1.8055514 | 0 |
| HALLMARK_MITOTIC_SPINDLE | 0.5862736 | 1.4523232 | 0 |
| HALLMARK_ESTROGEN_RESPONSE_LATE | 0.57552433 | 1.4265707 | 0.002450981 |
| HALLMARK_SPERMATOGENESIS | 0.5886695 | 1.3927197 | 0.015555556 |
| HALLMARK_MTORC1_SIGNALING | 0.5527332 | 1.3579533 | 0.00973236 |
| HALLMARK_GLYCOLYSIS | 0.52418345 | 1.285923 | 0.028571429 |
| HALLMARK_CHOLESTEROL_HOMEOSTASIS | 0.57865757 | 1.2706437 | 0.07366072 |
| HALLMARK_FATTY_ACID_METABOLISM | 0.51471084 | 1.2453758 | 0.039506175 |
| HALLMARK_MYC_TARGETS_V1 | 0.48373488 | 1.2021818 | 0.05121951 |
| HALLMARK_NOTCH_SIGNALING | 0.6193421 | 1.1901605 | 0.18818381 |
| HALLMARK_DNA_REPAIR | 0.47252923 | 1.1338636 | 0.14823009 |
| HALLMARK_HYPOXIA | 0.43898642 | 1.0890043 | 0.2116279 |
| HALLMARK_ESTROGEN_RESPONSE_EARLY | 0.4226998 | 1.0464121 | 0.30715936 |
| HALLMARK_PEROXISOME | 0.4538482 | 1.0346404 | 0.35866985 |
| HALLMARK_KRAS_SIGNALING_DN | 0.4184932 | 1.0319825 | 0.32943925 |
| HALLMARK_PROTEIN_SECRETION | 0.44562742 | 1.013377 | 0.389755 |
| HALLMARK_APICAL_SURFACE | 0.4962097 | 1.0008519 | 0.42236024 |
| HALLMARK_BILE_ACID_METABOLISM | 0.41916814 | 0.9714987 | 0.50577366 |
| HALLMARK_UV_RESPONSE_UP | 0.39980176 | 0.95552605 | 0.5808824 |
| HALLMARK_ADIPOGENESIS | 0.3828925 | 0.94748944 | 0.58128077 |
| HALLMARK_ANDROGEN_RESPONSE | 0.40898615 | 0.94509876 | 0.5518018 |
| HALLMARK_OXIDATIVE_PHOSPHORYLATION | 0.3814576 | 0.92956483 | 0.6627358 |
| HALLMARK_WNT_BETA_CATENIN_SIGNALING | 0.45552686 | 0.92251414 | 0.567033 |
| HALLMARK_HEME_METABOLISM | 0.36612138 | 0.9073999 | 0.7862233 |
| HALLMARK_XENOBIOTIC_METABOLISM | 0.36693737 | 0.90632516 | 0.7802691 |
| HALLMARK_HEDGEHOG_SIGNALING | 0.46300027 | 0.8906409 | 0.65052634 |
| HALLMARK_PI3K_AKT_MTOR_SIGNALING | 0.39056313 | 0.8878851 | 0.7408257 |
| HALLMARK_APOPTOSIS | 0.35960194 | 0.8721116 | 0.92081445 |
| HALLMARK_ANGIOGENESIS | 0.3647211 | 0.70372367 | 0.97940505 |
